# Exhaustive analysis and simple model of an angular displacement optical fiber sensor

**DOI:** 10.1038/s41598-025-05063-4

**Published:** 2025-06-03

**Authors:** Gorka Zubia, Joseba Zubia, Josu Amorebieta, Gotzon Aldabaldetreku, Gaizka Durana

**Affiliations:** 1 Department of Graphical Expression and Project Engineering, University of the Basque Country UPV/EHU, Bilbao, 48013 Spain; 2 Department of Communications Engineering, University of the Basque Country UPV/EHU, Bilbao, 48013 Spain; 3 EHU Quantum Center, University of the Basque Country UPV/EHU, Bilbao, 48013 Spain; 4 Department of Applied Mathematics, University of the Basque Country UPV/EHU, Bilbao, 48013 Spain

**Keywords:** Optical instrumentation, Optical fiber sensors, Intensity modulated optical sensor, Optical fiber displacement sensor, Photonic sensor, Structural health monitoring, Optical fiber devices, Optical metrology, Imaging and sensing, Fibre optics and optical communications, Optical sensors, Optoelectronic devices and components, Applied mathematics, Aerospace engineering, Electrical and electronic engineering

## Abstract

Accurate tilt-angle measurement is vital in applications ranging from aerospace to civil infrastructure monitoring, especially under harsh conditions where conventional inclinometers may fail. Here, we present a comprehensive analytical model for multi-axis tilt sensing based on intensity-modulated optical fiber sensors (OFDSs). By capturing how a Gaussian beam, reflected from a tilted target, couples into arrays of receiving fibers, our model bridges geometric fiber parameters, numerical aperture, and target distance to predict the measured power for various tilt angles and axes. We validate its performance experimentally using multiple fiber-bundle configurations: bifurcated, trifurcated, differential, symmetrical, and quasi–random 19-fiber arrangements, demonstrating accurate operation up to $\pm 20^\circ$ tilt over distances of up to 15 mm. In each case, the theoretical predictions match well with measured data, showing that differential or concentric fiber layouts suppress noise and eliminate ambiguities in tilt-direction detection. A $\pm 5\%$ parametric sweep shows that NA drift contributes $\le 6\%$ signal change, while core- and spacing-tolerances each add $< 4\%$, confirming that the sensor retains its specified accuracy when fabricated with standard-spec fibers. Compared to existing fiber-optic and mechanical inclinometers, our approach is simpler to fabricate, can be tailored to specific operational ranges, and remains reasonably resilient under the tested $\pm 5\%$ variations. Moreover, we show how multi-fiber geometries enable axis-wise tilt discrimination and improved sensitivity through differential measurements. These findings highlight the potential for cost-effective, real-time, multi-axis tilt sensors that can address Industry 5.0 and advanced physics lab instrumentation needs. Future work will extend the sensor to larger angular spans and complex reflective surfaces, aiming to further broaden its applicability and reach.

## Introduction

Industry 5.0 envisions a more human-centric, sustainable, and resilient future for manufacturing and automation, where advanced sensing and monitoring solutions are critical for efficient collaboration between humans and machines^1–3^. In this context, accurate tilt angle measurement is crucial in structural health monitoring and industrial automation, where even minor misalignments can compromise functionality and safety^4,5^. For instance, real-time blade tip clearance measurements in next-generation turbine engines rely on precise angle sensing to detect small tilt variations that may affect engine performance^6^. Similarly, monitoring tilt in civil infrastructure such as bridges and dams is indispensable for detecting early structural distress and preventing catastrophic failures^7,8^. However, many of these applications occur in harsh environments (high temperature, shock, or electromagnetic interference), posing significant challenges for conventional inclinometers like mechanical or MEMS-based tilt sensors^9^.

Optical fiber sensors (OFSs) offer a robust alternative under demanding conditions due to their immunity to electromagnetic interference, compact size, and remote-sensing capability^10,11^. Among optical tilt sensing methods, fiber Bragg grating (FBG) and Fabry–Pérot cavity sensors achieve sub-milliradian resolution but often require expensive interrogation setups and precise alignment^12,13^. In contrast, intensity-based optical fiber displacement sensors (OFDSs) provide a simpler, more cost-effective approach by monitoring the optical power reflected from a tilted target^14–21^. Despite these advantages, many intensity-based sensors rely on empirical calibrations and typically cannot differentiate tilt axes without multiple discrete configurations or additional hardware^6,7^.

Intensity-modulated optical fiber angular sensors (OFAS) have been studied for their advantages in lean angle measurement^22^ and angular displacement sensing^23^. Reflective OFDS have proven to be effective in detecting angular variations, as demonstrated by Wang et al. in the inspection of tilted objects^24^. The incorporation of grating reflectors has further enhanced their capability to measure multi-axis angles with high precision^25^. Geometrical parameter analysis of high-sensitivity OFAS has played a crucial role in optimizing sensor design and performance^26^. Both theoretical and experimental studies have contributed to improving the accuracy and stability of these sensors under different operating conditions^27^. Simulation and experimental studies on inclined two-fiber displacement sensors have provided valuable insights into sensor response characteristics^28,29^. Various fiber-optic sensor configurations, including twisted macro-bend coupling phenomena^30,31^, collimated beam systems, or integrated micro-displacement sensors, have been explored for enhanced angular measurement capabilities^4,32,33^. Furthermore, high-resolution fiber-optic sensors designed for precise angular displacement measurements have demonstrated their effectiveness in diverse applications such as the stroke of an electromagnetic actuator^34^, ultrasound detection^35^, automobile throttle position sensing^29^, modulators^36^ and surface slope^37^.

A key gap is the absence of a unified analytical framework that relates the tilt angle to the measured power in a multi-fiber arrangement. While some studies demonstrate single-axis tilt sensing with strong performance^5,8,38^ multi-axis sensing generally entails either multiple sensors or carefully designed mechanical linkages^15,39,40^. An accurate theoretical model, capable of predicting power variations for different tilt directions, would address this shortcoming and enable a versatile, low-cost multi-axis inclinometer.

In this work, we present a novel mathematical model for an OFDS configured to measure tilt angles about any orthogonal axes as a function of the fiber geometry. We have made an exhaustive evaluation of the model in a variety of fiber bundle configurations-symmetric, asymmetric and differential bifurcated, trifurcated and quasi-random, demonstrating how each feature-radii of the fibers, distance between them, spatial distribution of the fibers and NA affects responsivity, sensitivity, linearity and range of the sensor. We experimentally validated the model using both four commercial multi-fiber OFDSs and a custom-built trifurcated prototype, confirming its accuracy for tilt angles up to $\pm 15^\circ$ and target distances of up to 15 mm. Our trifurcated design allows for the simultaneous measurement of distance and angle. Subsequently, we compare our results with existing results from the literature.

Notably, this approach distinguishes tilt axes by analyzing the differential power among multiple receiving fibers, eliminating the need for separate calibration on each axis. Such a capability significantly expands the practical applicability of intensity-based sensors, offering robust and cost-effective angle measurements suitable for harsh industrial and aerospace environments^11^.

The paper is organized as follows. First, we derive the unified mathematical model. Next, we detail the experimental setup and procedures for validating the model. Subsequently, we compare our results with existing techniques from the literature. Finally, we discuss key applications and future research directions in multi-axis fiber-optic tilt sensing.

## Mathematical model for angular displacement

**Fig. 1:**
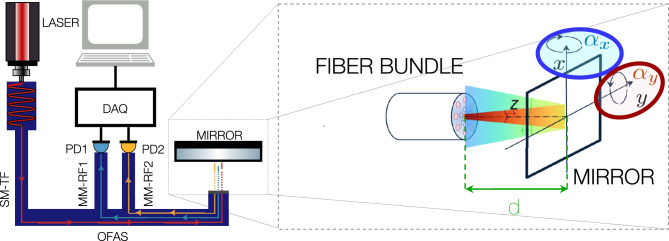
(**a**) Experimental setup of a typical OFAS. It consists of a laser light-source, an optical fiber bundle to guide the light and several photodectors (PDs), $\lbrace \text {PD}_1 \text {PD}_2,\dots \rbrace$, which convert the optical power into electrical signals and are quantized with a DAQ, and transmitted to a computer for signal processing. (**b**) Light propagation from the bundle tip.

The fundamental operational principle of an OFAS is the depicted in Fig. 1 and illustrates our overall measurement problem: a fiber bundle emmits light toward a target that can move along *z* and tilted by angles $\lbrace \alpha _x,\alpha _y\rbrace$. Sensor functionality requires capturing reflections at varying distances and orientations without requiring additional external optics. The beam coming from the transmitting fiber (TF) can be described by a Gaussian profile.

In^38^ the Gaussian beam profile was introduced in polar form. Here, we restate it in Cartesian form. If the total incident optical power is $P_0$ and the beam waist radius is^41^,

$$\begin{aligned} \omega (z)\approx z^2\tan ^2\theta _0\text {\qquad with } \textrm{NA} = \sin \theta _0 \end{aligned}$$

reformulating the irradiance *I*(*x*, *y*, *z*)^38,42^ at distance *z* is

1 $$\begin{aligned} I(z) \;=\; \frac{I_0}{\omega ^2(z)} \exp \!\Bigl (-\tfrac{2(x^2 + y^2)}{\omega ^2(z)}\Bigr ) \;=\; \frac{2\,P_0}{\pi \,\omega ^2(z)} \exp \!\Bigl (-\tfrac{2(x^2 + y^2)}{\omega ^2(z)}\Bigr ). \end{aligned}$$

### Effect of mirror tilt and coordinate transformation

**Fig. 2:**
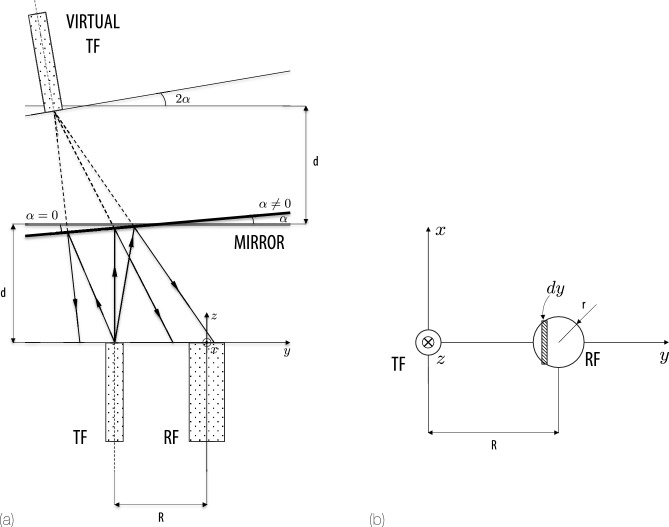
Definition of the geometrical parameters of the problem. *R* is the distance between the TF and the RF. *r* is the radius of the RF. $\alpha _x$ is the angle of the reflecting surface with respect to the *x*-axis. *d* is the distance between the fibers and the mirror for $\alpha _x=0^\circ$. (**b**) Integration procedure for the reflected signal on the RF.

The mirror is tilted by an angle $\alpha$ around the *x*-axis, creating angular and linear shifts in where the beam hits the receiving fiber (RF). To keep track of how the beam spot shifts when the mirror tilts, it is convenient to define a new coordinate system $\{x_R, y_R, z_R\}$ rotated by $2\alpha$ relative to $\{x,y,z\}$. This rotation by $2\alpha$ accounts for the fact that the reflected beam angle doubles in a mirror reflection scenario, see Fig. 2.

Let the center of the tilted mirror be located at *d* along the *y*-axis of the original coordinate system. Then, the transformation to the rotated system (around the x-axis) is given by

2 $$\begin{aligned} {\left\{ \begin{array}{ll} x_R \;=\; x, \\ y_R \;=\; y \cos 2\alpha \;-\; d \,\sin 2\alpha ,\\ z_R \;=\; d \;+\; d \cos 2\alpha \;+\; y\,\sin 2\alpha \;\approx \; d\,\Bigl (1 + \frac{1}{\cos 2\alpha }\Bigr ). \end{array}\right. } \end{aligned}$$

The expressions above capture how a point (*x*, *y*, *z*) in the original frame is viewed in the rotated frame. Note that throughout this mathematical model, we use $\alpha$ to denote a general tilt angle. However, in real-case geometries we may instead denote this angle as $\alpha _x$ or $\alpha _y$ , depending on whether the reflecting surface is tilted with respect to the x-axis or the y-axis, respectively. This distinction becomes important in later sections where we analyze specific sensor configurations. In this new rotated frame, the reflected beam arrives at the RF with an additional factor of $\cos 2\alpha$, that is, the projection of the rotated beam on the XY-plane. Consequently, the irradiance at the RF in the rotated coordinates is

3 $$\begin{aligned} I(z) \;=\; \frac{2\,P_0 \,\cos 2\alpha }{\pi \,\omega ^2(z_{R})} \exp \!\Bigl (-\dfrac{2(x^2 + y_R^2)}{\omega ^2(z_{R})}\Bigr ), \end{aligned}$$

where now,

$$\begin{aligned} \omega ^2(z_{R}) \;\approx \; d^2 \,(1+\cos (2\alpha ))^2 \,\frac{\tan ^2 \theta _0}{\cos ^2(2\alpha )}. \end{aligned}$$

### Total power collected

To find the total power collected by the RF, one integrates *I*(*z*) over the surface of the RF. Let the RF have a radius *r* and be located a distance *R* from the mirror rotation axis. The geometrical parameters are illustrated in Fig. 2 and listed on Table 1.

**Table 1:** Summary of the geometrical parameters of the problem depicted in Fig. 2.

Parameter	Meaning
$\alpha _x$	Angle of the reflecting surface w.r.t. the *x*-axis
*r*	Radius of the RF
*R*	Distance between the TF and the RF
*d*	Distance between the fibers and the mirror for $\alpha _x=0^\circ$

The received power is the double integral,

4 $$\begin{aligned} P_{R}(R,\alpha ,d) = \Gamma \iint _{R_x} I(x, y, z) \, dx \, dy \end{aligned}$$

whose limits are given by:

$$\begin{aligned} x \in \bigl [-\sqrt{r^2-(y-R)^2}, \sqrt{r^2-(y-R)^2}\bigr ], \quad y \in [ R-r, R+r ]. \end{aligned}$$

Then,

5 $$\begin{aligned} P_{R}(R,\alpha ,d) = \Gamma \dfrac{2P_o \cos ^3 2\alpha }{\pi \, d^2 \,\bigl (1 + \cos 2\alpha \bigr )^2 \,\tan ^2 \theta _0} \times \,\iint _{R_x}\exp \!\Bigl (-\dfrac{2\,x^2 \cos ^2 2\alpha }{d^2 \bigl (1 + \cos 2\alpha \bigr )^2 \tan ^2 \theta _0}\Bigr ) \, dx \nonumber \\ \times \,\iint _{R_x}\,\exp \!\Bigl (-\dfrac{2\,y_R^2 \cos ^2 2\alpha }{d^2 \bigl (1 + \cos 2\alpha \bigr )^2 \tan ^2 \theta _0}\Bigr )\, dy, \end{aligned}$$

where $R_x$ denotes the region of integration corresponding to the receiver area, and $\Gamma$ is the reflectivity of the mirror. Integrating with respect to *x* leads to

6 $$\begin{aligned} \int _{-\sqrt{r^2 - (y - R)^2}}^{\sqrt{r^2 - (y - R)^2}} \exp \!\Bigl (-\tfrac{2\,x^2 \cos ^2 2\alpha }{d^2 \bigl (1 + \cos 2\alpha \bigr )^2 \tan ^2 \theta _0}\Bigr ) \, dx= \int _{-\sqrt{r^2 - (y - R)^2}}^{\sqrt{r^2 - (y - R)^2}} \exp \!\bigl (-a x^2\bigr )\, dx= \frac{\sqrt{\pi } \,\textrm{erf}\!\bigl (\sqrt{a}\,\sqrt{r^2 - (y - R)^2}\bigr )}{\sqrt{a}}, \end{aligned}$$

where $\textrm{erf}(\cdot )$ is the error function and

$$\begin{aligned} a \;=\; \frac{2 \cos ^2(2\alpha )}{ d^2 \,\bigl (1+\cos 2\alpha \bigr )^2 \,\tan ^2 \theta _0}. \end{aligned}$$

The remaining integral becomes

7 $$\begin{aligned} \int _{R-r}^{R+r} \frac{\sqrt{\pi } \,\textrm{erf}\!\Bigl (\sqrt{a}\sqrt{r^2 - (y - R)^2}\Bigr )}{\sqrt{a}}\, \exp \!\Bigl (-a y_R^2\Bigr ) \, dy= \nonumber \\ = \sqrt{\frac{\pi }{a}} \int _{R-r}^{R+r} \textrm{erf}\!\Bigl (\sqrt{a}\sqrt{r^2 - (y - R)^2} \Bigr ) \exp \!\Bigl (-a(y\cos 2\alpha - d\sin 2\alpha )^2\Bigr ) \, dy. \end{aligned}$$

### Approximate solution and detected power

The integral derived in Eqs. (6) and (7) can be evaluated numerically. However, it is often more convenient to approximate it in order to see explicitly how the detected power depends on different parameters. By applying the mean value theorem for integrals, we approximate the value of the integral by evaluating the irradiance at the center of the RF, i.e. at $y = R$. Thus, we rewrite Eq. (7):

8 $$\begin{aligned} 2r \,\sqrt{\frac{\pi }{a}} \,\text {erf}\bigl (\sqrt{a}\,r\bigr ) \exp \!\Bigl (-a\bigl (R\cos 2\alpha - d\sin 2\alpha \bigr )^2\Bigr ). \end{aligned}$$

With this approximation, and expanding *a*, the power received by the RF is described by,

9 $$\begin{aligned} P_{R_i}&= \underbrace{ \overbrace{ \frac{2\sqrt{2}\,r\,\Gamma \,P_0\,\cos ^2(2\alpha )}{\sqrt{\pi }\,d\,(1+\cos 2\alpha )\,\tan \theta _0} }^{\text {prefactor}} \;\times \; \overbrace{ \textrm{erf}\!\Bigl ( \frac{\sqrt{2}\,r \,\cos 2\alpha }{d\,(1 + \cos 2\alpha )\,\tan \theta _0} \Bigr ) }^{\text {error function}} }_{f(r,\alpha ,d)} \;\times \; \underbrace{ \overbrace{ \exp \!\Bigl ( -\,\frac{2\,\bigl (R\cos 2\alpha - d\sin 2\alpha \bigr )^2 \cos ^2(2\alpha )}{d^2\,(1 + \cos 2\alpha )^2\,\tan ^2 \theta _0} \Bigr ) }^{\text {Gaussian-type suppression}} }_{g(R,\alpha ,d)} \,. \end{aligned}$$

Observe that the part involving *r* appears in front- including the error function- while the part involving *R* appears inside the exponential. That motivates us to factor it into two functions, $f\bigl (r,\alpha ,d\bigr )$ and $g\bigl (R,\alpha ,d\bigr )$. We now write in a more compact form.

10 $$\begin{aligned} P_{R_i} \;=\; f\bigl (r,\alpha ,d\bigr )\,\times \,g\bigl (R,\alpha ,d\bigr ). \end{aligned}$$

From this point on, we will assume that the reflectivity of the target is one, i.e. $\Gamma =1$. In the more general case of a rough surface, the reflection coefficient $\Gamma$ in the equations would be less than unity. Since $\Gamma$ multiplies every term in Eqs. (4)–(10), its variation does not distort the exponential or error-function terms governing the dependence on distance *d* and angle $\alpha$; rather, it uniformly scales the entire response curve up or down. Consequently, the signal-to-noise ratio decreases as less reflected light is coupled back into the RF.

**Fig. 3:**
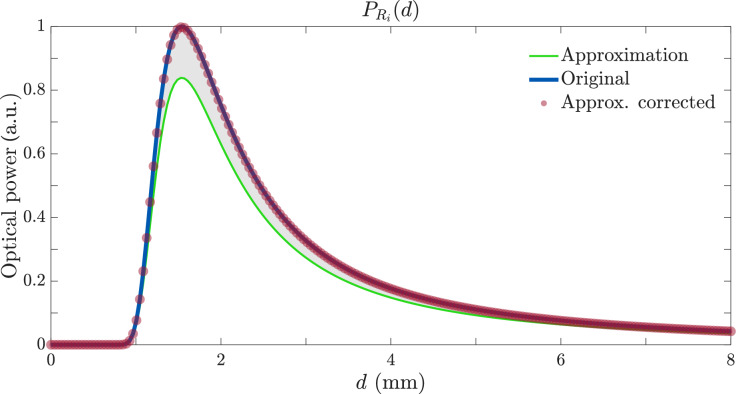
Power received by the RF of a bifurcated bundle as a function of distance *d* with $\text {NA}=0.09$, $r=50$ µm, $R=600$ µm, and $\alpha = 0^\circ$. The blue curve depicts the response according to Eq. (7), the green, the approximation of Eq. (9), and the red is approximation of Eq. (9) scaled with a prefactor of 1.2.

Figure 3 illustrates the response of a bifurcated bundle (one TF and one RF) as a function of the distance *d* for $\text {NA}=0.09$, $r = 50$ µm, $R = 600$ µm, and $\alpha = 10^\circ$. We observe that the analytic approximation of the integral is quite accurate, exhibiting the same qualitative behavior as the exact numerical result, thus, it is also highly precise. Consequently, for the remainder of this chapter, we adopt the simpler closed-form expression in Eq. (9) when computing the power received by the RF. The theoretical model is not limited to silica fibers and it can used with high–NA fibers, like polymer optical fibers, by adjusting the beam divergence angle $\theta _0$ in Eq. (9). The model assumes a Gaussian beam profile emerging from the fiber.

### Limitations of the mean-value approximation

Equation (4) is obtained by applying the mean-value theorem to the *y*-integration of the exact Eq. (7) after the inner *x*-integral has been carried out analytically. Doing so introduces a constant scale bias (the 1.2 factor in Fig. 3), and a residual *shape* error.

The leading constant bias arises from evaluating the inner integral at $y = R$ rather than averaging over $y \in [R - r, R + r]$; this bias is eliminated through the single-point calibration performed in every experiment. After that, the residual shape error can be quantified by a second-order Taylor expansion around the fiber center $y = R$ (see the Appendix), yielding the error bound:

11 $$\begin{aligned} \varepsilon (d,\alpha ) = \frac{\bigl |P_{\textrm{approx}} - P_{\textrm{exact}}\bigr |}{P_{\textrm{exact}}} \le \frac{r^2}{6}\!\Bigl (\frac{4R^2}{\omega ^4} - \frac{2}{\omega ^2}\Bigr ) + {\mathcal {O}}\!\bigl (\tfrac{r^4}{\omega ^4}\bigr ) \end{aligned}$$

Within the normal operating window ($d \le 10\ \textrm{mm}$, $|\alpha | \le 20^\circ$, $\textrm{NA}\ge 0.2$), this predicts $\varepsilon _{\max }=1.8\%$, translating to $0.2^\circ$ angular uncertainty. At very short distances or for low-divergence beams ($\textrm{NA}<0.15$), the term $r^2/\omega ^2$ grows, and the error exceeds 5%. Conversely, for high-NA POFs ($0.25 \le \textrm{NA} \le 0.45$), a larger NA broadens the far-field spot, reducing $\varepsilon$ to 0.9% at $\textrm{NA}=0.45$.

The approximation error is related to the intensity gradient across the RF core. Since a higher $\textrm{NA}$ corresponds to larger $\theta _0$ and thus larger $\omega (d,\alpha )$, the reflected spot covers the RF more uniformly, improving approximation accuracy as $\textrm{NA}$ or distance increases. Specifically, since $\varepsilon \propto 1/\omega ^2$, the error decreases monotonically with $|\alpha |$. To second order,

12 $$\begin{aligned} \varepsilon (\alpha )\approx 1-4\alpha ^2, \end{aligned}$$

indicating the worst error (1.8%) at $\alpha =0^\circ$, dropping by $\approx 25\%$ at $10^\circ$ and 40% by $20^\circ$. This is symmetric, $\varepsilon (+\alpha )=\varepsilon (-\alpha )$.

Although $\varepsilon$ scales with $r^2$, the tight dimensional tolerances of telecom–grade fibers ensure this effect remains modest and easily accommodated by the single-point calibration already performed. For example, a $\pm 10\%$ variation in $r$ results in only a $\pm 20\%$ change in $\varepsilon$.

Practically, this implies the mean-value approximation remains accurate and reliable within typical operating conditions, provided careful calibration is performed. Future enhancements could incorporate higher-order approximations or numerical integration methods to further reduce errors, especially for applications involving low-NA fibers or critical short-distance measurements.

## Results

In this section, we examine the implications of the proposed model. We begin with the fundamental bifurcated topology then proceed to a symmetric configuration featuring two RFs. Finally, we explore additional differential approaches that employ multiple RFs to further elucidate the versatility of this model.

### Bifurcated bundle

**Fig. 4:**
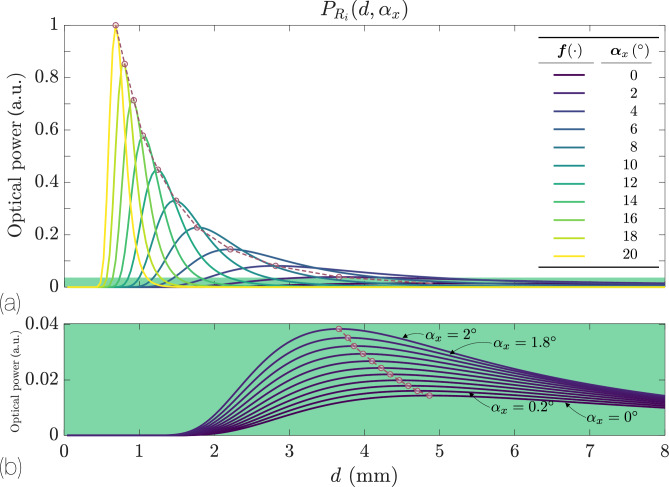
Power received by the RF of a bifurcated bundle as a function of distance for different angles, according to Eq. 9. The red dots indicate the position of the maximum $d_{\text {max}}$ of the power $P_{R_{i}}$. The dashed red line is the formant of those maxima. (**a**) Here, $\text {NA}=0.09$, $r=50$ µm, $R=600$ µm, and $\alpha _x\in \lbrace 0,20\rbrace ^\circ$. (**b**) Zoom of the response from $\alpha _x = 0^\circ$ to $2^\circ$ every $0.2^\circ$.

Figure 4 shows a representative response of a bundle comprising one TF and one RF, following the configuration depicted in Figure 2b. For this particular example, the parameters are $\text {NA}=0.09$, $r = 50$ µm, $R = 600$ µm, and a tilt angle $\alpha _x\in \lbrace 0,20\rbrace ^\circ$ every of $2^\circ$, see Fig. 4a. Figure 4b highlights the response in the narrower range of $0^\circ$ to $2^\circ$ with finer increments of $0.2^\circ$. The detected power increases significantly as the reflective surface is tilted. Quantitatively, the power received at a tilt angle of $20^\circ$ is approximately 60 times greater than at $0^\circ$. However, the response of this bifurcated bundle is *bivalued*; namely, the same detected power corresponds to two distinct distance values.

This complicates its use in sensor designs unless the operating range is restricted to one of the two response regions- the front slope or the back slope. As illustrated in Fig. 4b, the response of the bundle is also appreciable for small tilt angles.

It is also observed that for a bifurcated bundle, as the tilt angle $\alpha _x$ increases, the response curve shifts to shorter distances. The distance at which maximum response occurs can thus be used to detect the surface angle, as it is uniquely defined. Indeed, the envelope of these maxima readily generates a calibration curve, shown with the dashed red line joining the red scatter points.

The appearance of these maxima is straightforward. As the angle $\alpha _x$ increases, the peak of the reflected Gaussian beam shifts to progressively larger values of *y*. When the condition $\tan 2\alpha _x = R / d_{\text {max}}$ is satisfied, the irradiance maximum is located exactly at the center of the RF. For a fixed fiber separation *R*, the position of this maximum $d_{\text {max}}$ is pushed to larger values as $\alpha _x$ decreases. An opposite effect is observed as *R* increases: $d_{\text {max}}$ also increases.

Another noteworthy aspect is the strong asymmetric behavior of the response with respect to $\alpha _x$. At equal angles of opposite sign- e.g., $\pm \alpha _x$- the response is much smaller for negative angles. Consequently, a bifurcated bundle cannot efficiently detect both positive and negative rotations. To remedy this limitation, a symmetric fiber arrangement is required.

**Fig. 5:**
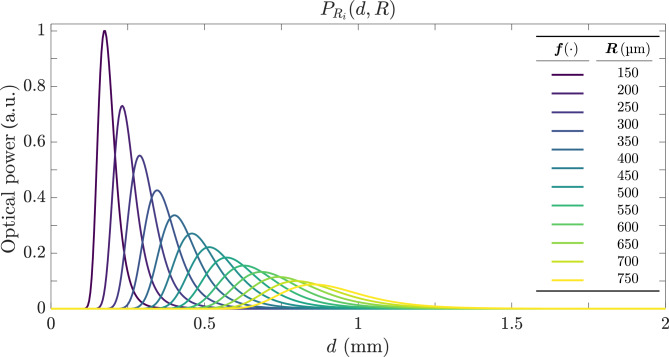
Power received by the RF of a bifurcated bundle according to Eq. 9 as a function of distance *d*, for various values of *R* ranging from 150 µm (blue) to 750 µm (orange) in increments of 50 µm, with $\alpha _x=20^\circ$, $\text {NA}=0.09$, and $r=50$ µm.

A final observation concerns the relationship between the inclination angle and the measurement range of the system. Larger tilt angles $\alpha _x$ produce a more peaked response with higher amplitude, shifting to shorter distances and offering increased sensitivity but reduced range. Bringing the TF and RFs closer together greatly boosts sensitivity but drastically shrinks the operating distance of the sensor. Figure 5 illustrates this behavior for a bifurcated bundle model at $\alpha _x=20^\circ$, with $\text {NA}=0.09$, $r=50$ µm, and *R* ranging from 150 µm (blue) to 750 µm (orange) in steps of 50 µm. In Secs. Symmetric bifurcated bundle and Differential bundle we analyze two variants of the bifurcated bundle designed to improve the response symmetry, and to reduce its susceptibility to noise.

### Symmetric bifurcated bundle

**Fig. 6:**
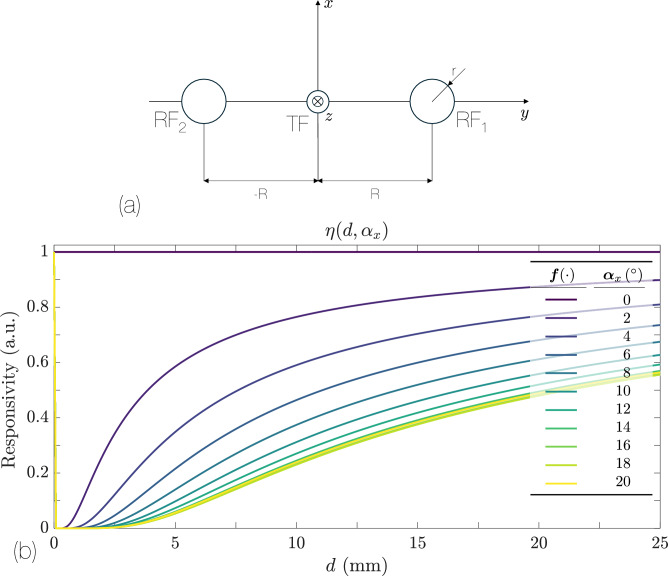
(**a**) Geometry of a symmetric bifurcated bundle, featuring two RFs at $+R$ and $-R$. (**b**) Detected power ratio $\eta (\alpha _x,d)$ according to Eq. (15) as a function of the distance *d*, for different values of $\alpha _x$ ranging from $0^\circ$ to $20^\circ$ in increments of $2^\circ$. The parameters are $\theta _o = 12.71^\circ$, $r = 50$ µm, and $R = 600$ µm.

A straightforward way to achieve a more balanced response to positive and negative tilt angles is to use two identical RFs placed symmetrically about the TF, one at a distance $+R$ and the other at $-R$. Figure 6a illustrates this geometry. The power collected by each of the two RFs is given by:

13 $$\begin{aligned} P_{R_1}(R)&= \frac{2\sqrt{2}\,r\,P_0 \,\cos ^2 2\alpha _x}{\sqrt{\pi }\,d\,\bigl (1 + \cos 2\alpha _x\bigr )\,\tan \theta _0} \,\text {erf}\!\Bigl (\frac{\sqrt{2}\,r\,\cos 2\alpha _x}{d\,\bigl (1 + \cos 2\alpha _x\bigr )\,\tan \theta _0}\Bigr ) \nonumber \\&\quad \times \exp \!\Bigl (-\dfrac{2\,\cos ^2 2\alpha _x\,\bigl (R\cos 2\alpha - d\sin 2\alpha _x\bigr )^2}{d^2\,\bigl (1 + \cos 2\alpha _x\bigr )^2\,\tan ^2 \theta _0}\Bigr ), \end{aligned}$$

14 $$\begin{aligned} P_{R_2}(-R)&= \frac{2\sqrt{2}\,r\,P_0 \,\cos ^2 2\alpha _x}{\sqrt{\pi }\,d\,\bigl (1 + \cos 2\alpha _x\bigr )\,\tan \theta _0} \,\text {erf}\!\Bigl (\frac{\sqrt{2}\,r\,\cos 2\alpha _x}{d\,\bigl (1 + \cos 2\alpha _x\bigr )\,\tan \theta _0}\Bigr ) \nonumber \\&\quad \times \exp \!\Bigl (-\dfrac{2\,\cos ^2 2\alpha _x\,\bigl (-R\cos 2\alpha _x - d\sin 2\alpha _x\bigr )^2}{d^2\,\bigl (1 + \cos 2\alpha _x\bigr )^2\,\tan ^2 \theta _0}\Bigr ). \end{aligned}$$

We now define the responsivity $\eta (\alpha _x, d)$ as the ratio of the output voltages in the two PDs:

15 $$\begin{aligned} \eta (\alpha _x, d)&= \frac{V_2}{V_1} \;=\; \frac{k_2 \,P_{R_2}(-R)}{k_1 \,P_{R_1}(R)} \;=\; \frac{\exp \Bigl (-\tfrac{2\,\cos ^2 2\alpha _x\,\bigl (-R\cos 2\alpha _x - d\sin 2\alpha _x\bigr )^2}{d^2\,\bigl (1 + \cos 2\alpha _x\bigr )^2\,\tan ^2 \theta _0}\Bigr )}{\exp \Bigl (-\tfrac{2\,\cos ^2 2\alpha _x\,\bigl (R\cos 2\alpha _x - d\sin 2\alpha _x\bigr )^2}{d^2\,\bigl (1 + \cos 2\alpha _x\bigr )^2\,\tan ^2 \theta _0}\Bigr )} = \exp \!\Bigl ( -\dfrac{8\,R\,\sin 2\alpha _x\,\cos ^3 2\alpha _x}{d\,\bigl (1 + \cos 2\alpha _x\bigr )^2\,\tan ^2 \theta _0} \Bigr ), \end{aligned}$$

where $V_i$ are the voltages at each photodetector (PD), and $k_i$ are proportionality constants- gains, noise factors, etc. We assume identical PDs with the same gain, so $k_1 = k_2$, which greatly simplifies Eq. (15) above.

Compared to a simple bifurcated bundle, one of the main advantages of this symmetric configuration is that the ratio $\eta (\alpha _x, d)$ in Eq. (15) cancels out common noise terms, yielding improved performance. Since the fibers are arranged symmetrically, the angular response is the same for $\alpha _x$ and $-\alpha _x$, which means that the sensor can detect rotations in both senses of the same direction. Moreover, for the symmetric arrangement in Fig. 6b the response is monotonically increasing with distance for a given $\alpha _x$, though the amplitude of the ratio decreases with $\alpha _x$. This behavior effectively linearizes the response of the sensor but also reduces its overall sensitivity compared to a single RF design. From a practical standpoint, however, the trade-off can be beneficial: the sensor is more insensible to variations in $\alpha _x$ and can detect any inclination of the reflective surface accurately.

Taking the natural logarithm of Eq. (15) gives:

16 $$\begin{aligned} \ln \eta (\alpha _x,d) \;=\; \frac{8\,R\,\sin 2\alpha _x\,\cos ^3 2\alpha _x}{d\,\bigl (1 + \cos 2\alpha _x\bigr )^2\,\tan ^2 \theta _0}\approx \frac{8R}{d\tan ^{2}\theta _0}\left( 0.095\sin 5.2\alpha _x\right) . \end{aligned}$$

**Fig. 7:**
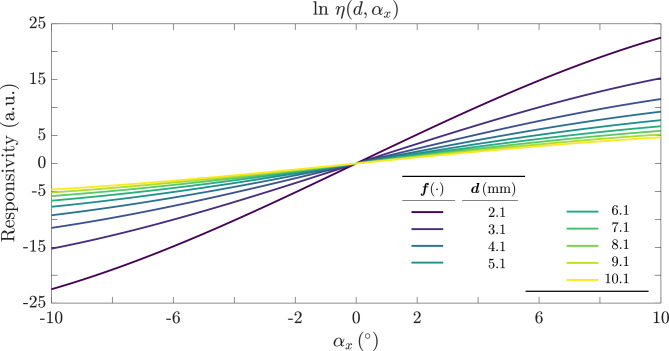
Natural logarithm of the responsivity for $d = 2.1\,\textrm{mm}$- steepest slope- to $10.1\,\textrm{mm}$- least steep- in steps of $1\,\textrm{mm}$. The parameters are $\text {NA}=0.09$, $\lbrace r_1 = r_2 =50,R =600\rbrace$ µm. We can appreciate the nearly linear dependence of the responsivity with respect to $\alpha _x$, distinguishing positive from negative angles.

Figure 7 shows the natural logarithm of $\eta (\alpha _x, d)$ versus the angle $\alpha _x$, for different values of the distance *d*. The relationship is fairly linear with respect to $\alpha _x$, and it is symmetric for positive and negative angles. In this plot, $\theta _o = 5^\circ$, $\lbrace r_1 = r_2 =50,R =600\rbrace$ µm, but note that the fiber radius *r* does not enter into $\eta (d,\alpha _x)$. This occurs because the approximation factorizes the dependence on *r*, see (9).

### Differential bundle

**Fig. 8:**
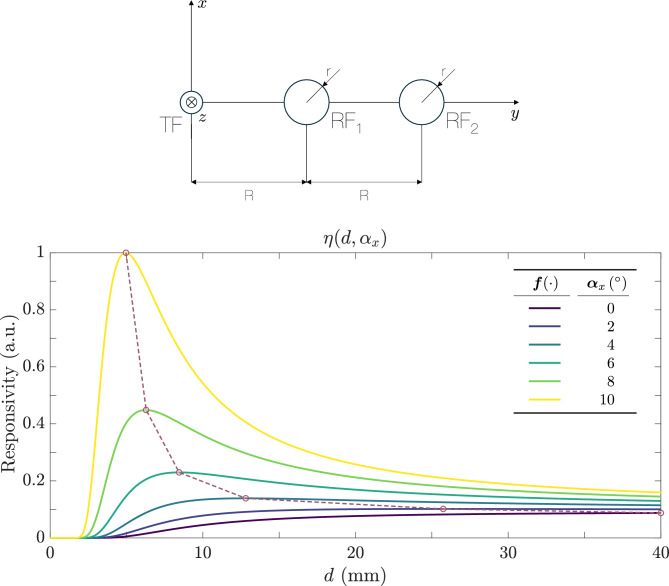
(**a**) Configuration of a differential trifurcated bundle. The TF is at the center, one RF is at *R*, and the other at 2*R*. (**b**) Responsivity $\eta (\alpha _x, d)$ according to Eq. (17), as a function of the distance *d* for different angles $\alpha _x$ ranging from $0^\circ$ (blue) to $10^\circ$ (red) in $2^\circ$ increments. The parameters are $\theta _o = 5^\circ$, $r = 50$ µm, and $R=600$ µm.

In this configuration, two fibers are placed on the same side with respect to the TF. As a particular case study, we assume that the RFs are identical and fixed at distances *R* and 2*R* from the TF. Figure 8 illustrates this arrangement. Starting from Eq. (9), we define the responsivity for this trifurcated bundle as

17 $$\begin{aligned} \eta (\alpha _x, d)&= \frac{P_{R_2}(2R)}{P_{R_1}(R)} = \frac{\exp \Bigl (-\tfrac{2\,\cos ^2 2\alpha _x\;\bigl (2R\cos 2\alpha _x - d\sin 2\alpha _x\bigr )^2}{d^2\,\bigl (1 + \cos 2\alpha _x\bigr )^2\,\tan ^2 \theta _o}\Bigr )}{\exp \Bigl (-\tfrac{2\,\cos ^2 2\alpha _x\;\bigl (R\cos 2\alpha _x - d\sin 2\alpha _x\bigr )^2}{d^2\,\bigl (1 + \cos 2\alpha _x\bigr )^2\,\tan ^2 \theta _o}\Bigr )} = \exp \!\Bigl ( -\dfrac{2R\,\cos ^3 2\alpha _x\,\bigl (3R\cos 2\alpha _x - 2d\sin 2\alpha _x\bigr )}{d^2\,\bigl (1 + \cos 2\alpha _x\bigr )^2\,\tan ^2 \theta _o} \Bigr ). \end{aligned}$$

Figure 8 also shows how the displacement response of the sensor depends on the tilt angle $\alpha _x$.

Compared to a simple bifurcated bundle, the differential arrangement also benefits from noise cancellation through the ratio in Eq. (17), thus improving measurement robustness. As $\alpha _x$ increases from $0^\circ$ to $10^\circ$, the amplitude of the responsivity increases. However, unlike bifurcated bundles, the maximum appears at angles different from zero. This feature is important and distinctive of these bundles, allowing us to identify the rotation of the target. If a maximum appears in the response, the bundle tip is not aligned perpendicularlly with respect to the target (mirror); in other words, the latter is rotated.

### Trifurcated bundle

**Fig. 9:**
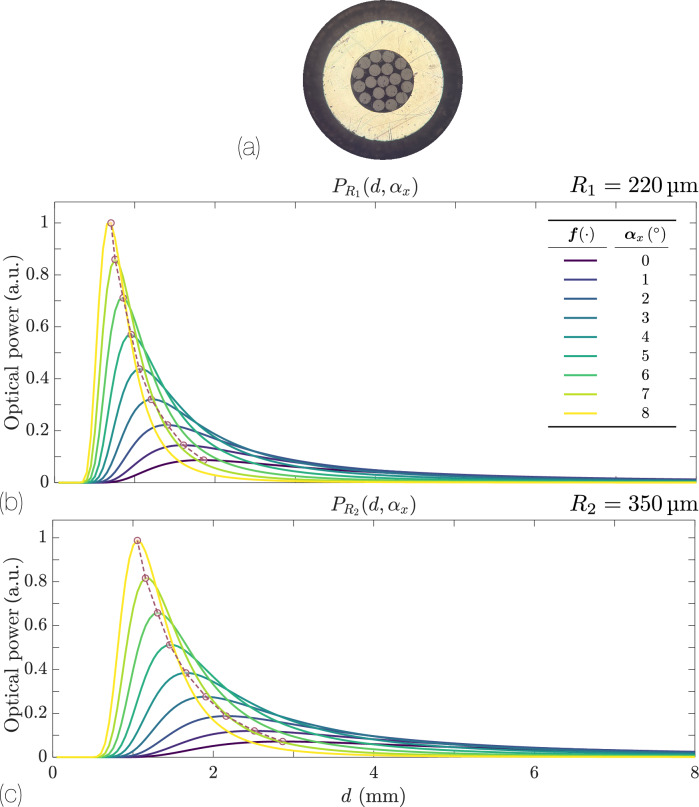
(**a**) Bundle tip of our custom designed and manufactured trifurcated bundle. It consists of a TF and 18 identical RFs arranged in two collections, of 6 and 12 RFs respectively. (**b**) Power collected by the 1$^\text {st}$ RF collection $\lbrace R_1=220$ µm, $N_1 =6\rbrace$ for different tilt angles with $r=100$ µm and $\text {NA}=0.22$. (**c**) Same as (**b**) but for the 2$^\text {nd}$ RF collection: $R_2=350$ µm, $N_2=12$.

This configuration is an azimuthal extension of the differential bundle: instead of having just two RFs at different distances from the TF, the bundle is formed by two concentric RFs collections as shown in Fig. 9a. The power collected by each RF collection is,

18 $$\begin{aligned} P_{R_i}(z) \approx \frac{2\,N_i\,\sqrt{2}\,r_i\,P_0\,\cos ^2 2\alpha _x}{\sqrt{\pi }\,d\,\bigl (1 + \cos 2\alpha _x\bigr )\,\tan \theta _o} \,\text {erf}\!\Bigl (\frac{\sqrt{2}\,r_i\,\cos 2\alpha _x}{d\,\bigl (1 + \cos 2\alpha _x\bigr )\,\tan \theta _o}\Bigr ) \exp \!\Bigl (-\dfrac{2\,\cos ^2 2\alpha _x\,\bigl (R_i \cos 2\alpha _x - d \sin 2\alpha _x\bigr )^2}{d^2\,\bigl (1 + \cos 2\alpha _x\bigr )^2\,\tan ^2 \theta _o}\Bigr ), \end{aligned}$$

where $N_i$ is the number of RFs at $R_i$ from the TF center, and $r_i$ is the radii of the RFs.

Figures 9b and c show the responses of the two collections, calculated from Eq. (18), for $\text {NA}=0.22$, $r=100$ µm, and thus $\theta _0=12.7^\circ$. The inner RF collection $\lbrace R_1=220$ µm, $N_1=6\rbrace$ is closer to the transmitter than the outer RF collection $\lbrace R_2=350$ µm, $N_2=12\rbrace$. Because the outer RF collection is farther away, its collected power peaks at larger distances *d* and has a generally wider (but lower-amplitude) response. In particular, the dead zone- the range of distances where the collected power is below 5% of its maximum- grows larger with increasing $\text {TF}-\text {RF}$ separation^17,38,41^. As in previous cases, we define a differential responsivity $\eta (\alpha _x, d)$ from the ratio of the powers detected by the inner and outer RF collections:

19 $$\begin{aligned} \eta (\alpha _x, d)&\;=\; \frac{P_{R_2}\bigl (R_2\bigr )}{P_{R_1}\bigl (R_1\bigr )} \;\approx \; \frac{N_2}{N_1} \exp \!\Bigl (-\dfrac{2\,(R_2 - R_1)\,\cos ^3 2\alpha _x\,\bigl ((R_2 + R_1)\cos 2\alpha _x - 2\,d\,\sin 2\alpha _x\bigr )}{d^2\,\bigl (1 + \cos 2\alpha _x\bigr )^2\,\tan ^2 \theta _o}\Bigr ), \end{aligned}$$

**Fig. 10:**
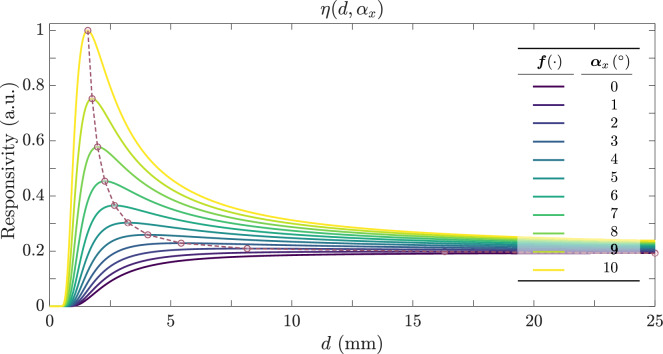
Responsivity $\eta (\alpha _x,d)$ vs. distance *d* for various tilt angles $\alpha _x$ from $0^\circ$- light blue, lowest amplitude- to $10^\circ$- red, highest amplitude- in $1^\circ$ increments. The setup parameters $\text {NA}=0.22$ and $\lbrace r=100, R_1=220, R_2=350\rbrace$ µm match those in Fig. 9.

Figure 10 shows $\eta (\alpha _x,d)$ for angles $\alpha _x\in [0,10]^\circ$. Notably, this concentric fiber geometry yields a response that is independent of the specific azimuthal orientation of the reflecting surface tilt (orientation of the x-axis); it behaves similarly for any rotation axis orthogonal to *z*. Moreover, the presence of a maximum in $\eta (\alpha _x,d)$ can be exploited to determine whether the surface is tilted and by how much, isolating the tilt $\alpha _x$ and distance *d* contributions. A trade-off emerges when $\eta (\alpha _x,d)$ is *bivalued* with respect to *d* (except for $\alpha _x=0^\circ$). Although this complicates direct range measurements, it allows one to infer $\alpha _x$. For instance, taking $R_2=2R$ and $R_1=R$, there exist two distance values $d_1\ne d_2$ such that

20 $$\begin{aligned} \eta (\alpha _x,d_1) \;=\; \eta (\alpha _x,d_2), \end{aligned}$$

then straightforward algebra from Eqs. (18) and (19) shows that,

21 $$\begin{aligned} \tan 2\alpha _x \;=\; \frac{3R}{2\,d_1\,d_2}\,(d_1 + d_2), \end{aligned}$$

which provides a direct expression for $\alpha _x$. Therefore, while bivalued responsivities can limit direct measurement of the distance *d*, they also create a new route for measuring the tilt angle $\alpha _x$ of the reflecting surface.

### Randomly distributed 19-fiber bundle

**Fig. 11:**
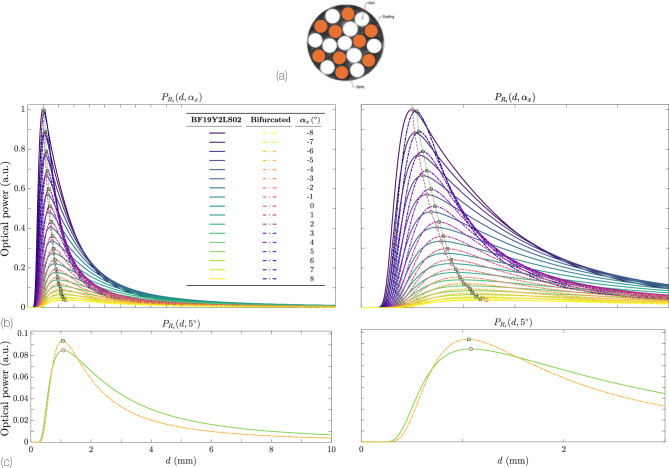
(**a**) Geometry of a bifurcated 19-fiber bundle (BF19Y2LS02). The nine TFs are shown in white, and the ten RFs in black. (**b**) Comparison of the BF19Y2LS02 detected power and a simple bifurcated bundle with two fibers of the same radius and separated by $R = 245$ µm. It shows $\alpha _x\in \lbrace -8,+8\rbrace ^\circ$. A scale factor of 11 was applied to the bifurcated bundle response. (**c**)  Same as (**b**) but just for an angle $\alpha _x=5^\circ$. The NA of all fibers is 0.22. Left figures correspond to $d\in \lbrace 0,10\rbrace$ mm, and the right ones to $d\in \lbrace 0,3\rbrace$ mm.

Let us now consider a bundle in which about half of the fibers- nine fibers shown in white- serve as TFs, while the other ten are RFs- shown in black. Figure 11 depicts this arrangement. The total power collected by all RFs is found by summing the individual contributions from each TF, taking into account the various $\text {TF}-\text {RF}$ distances. Formally,

22 $$\begin{aligned} \eta (\alpha _x, d)&= \sum _{i=1}^{10} \sum _{\begin{array}{c} j=1 \\ i \ne j \end{array}}^9 P_{R_i}\bigl (R_i - R_j\bigr ) = \frac{2\sqrt{2}\,r\,P_0\,\cos ^2 2\alpha _x}{\sqrt{\pi }\,d\,\bigl (1 + \cos 2\alpha _x\bigr )\,\tan \theta _0} \,\text {erf}\!\Bigl (\dfrac{\sqrt{2}\,r\,\cos 2\alpha _x}{d\,\bigl (1 + \cos 2\alpha _x\bigr )\,\tan \theta _0}\Bigr ) \nonumber \\&\quad \times \sum _{i=1}^{10} \sum _{\begin{array}{c} j=1 \\ i \ne j \end{array}}^9 \exp \!\Bigl (-\dfrac{2\,\cos ^2 2\alpha _x\,\bigl (\sqrt{(x_i - x_j)^2 + (y_i - y_j)^2}\,\cos 2\alpha _x - d\,\sin 2\alpha _x\bigr )^2}{d^2\,\bigl (1 + \cos 2\alpha _x\bigr )^2\,\tan ^2 \theta _0}\Bigr ), \end{aligned}$$

where $\bigl (x_i,y_i\bigr )$ and $\bigl (x_j,y_j\bigr )$ represent the positions of the RFs and TFs in the plane perpendicular to the optical axis. The core radii of all the fibers are identical, $r_i = r\,\forall \, i$.

Figure 11b compares the detected power for the commercial randomly distributed 19-fiber bundle (BF19Y2LS02, Thorlabs, Newton, NJ, US), red curves, to that of a simpler bifurcated arrangement with two fibers of identical radius, blue, separated $R=245$ µm. In both cases, $\alpha _x$ spans from $-8^\circ$ to $+8^\circ$. Despite sharing a common maximum point, the many-fiber bundle offers an advantage at larger distances. This is because the BF19Y2LS02 includes multiple $\text {TF}-\text {RF}$ separations. As we have seen, having a spread of fiber separations increases the overall distance range over which a significant response is measured^41^.

### Sensitivity analysis

A practical sensor must tolerate the normal dimensional spread of telecom–grade fibers (core $\pm 2\%$, NA $\pm 3\%$, position $\pm 5\%$). To quantify that tolerance we have evaluated the analytical transfer–functions for all five bundle layouts and vary one parameter at a time by $\pm 5\%$ around the nominal silica values ($r=50$ µm, $\textrm{NA}=0.22$, $R=600$ µm, $R_1=220$ µm, $R_2=350$ µm). Because the approximation error depends on tilt, we take the sensitivities at $\alpha =0^\circ$,where the power error is largest, and again at $\alpha =10^\circ$.

For the basic bifurcated bundle, a $-5\%$ drift in $\textrm{NA}$ causes a $-5.8\%$ power change; $\pm 5\%$ in $r$ or $R$ moves the signal by $\le 3.3\%$. Symmetric and differential bundles cancel the radius term entirely, and the differential bundle keeps every tolerance below $4\%$ at $\alpha =10^\circ$, confirming its reliability. The trifurcated ratio is nearly NA–independent at $\alpha =0^\circ$ and its sensitivity grows at large tilts ($>10^\circ$), but still the drift is under $\pm 11\%$. Even this worst-case translates to $<0.3^\circ$ angle error, so standard telecom fibers ($\textrm{NA}\pm 3\%$, core $\pm 2\%$) remain sufficient and more stringent tolerances are unnecessary.

## Model validation: experimental setup

**Fig. 12:**
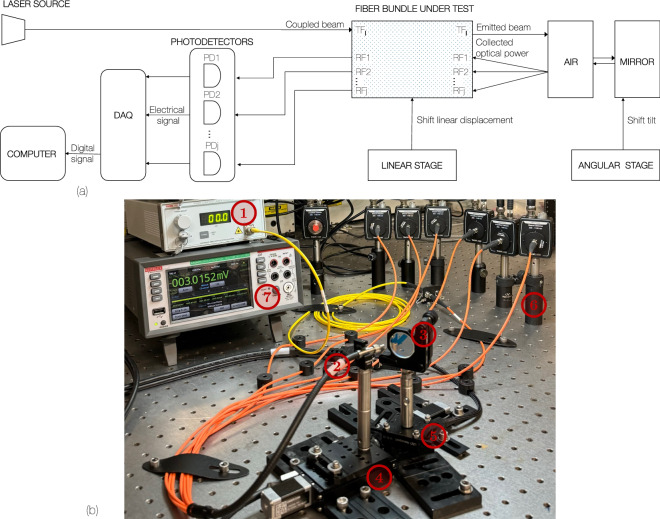
Typical optical fiber angular sensor (OFAS). (**a**) Schematic diagram of the experimental setup. (**b**) Experimental setup: (1) a 660 nm laser source, (2) the fiber bundle, and (6) several quadratic photodetectors $\text {PD}_i$, which convert optical power into electrical signals. These signals are digitized by (7) a DAQ for further processing. The other components are (3) a mirror, (4) a linear and (5) an angular displacement stages.

To validate the theoretical model presented in Sec. 2, we designed the experimental arrangement shown in Fig. 12. It consists of seven main elements (1) A 660 nm Fabry-Perot benchtop laser source (S4FC660, Thorlabs, Newton, NJ, US), (2) the fiber bundle under test, (3) mirror serving as the reflective target, (4) a linear (X-LSM025A, Zaber, Vancouver, British Columbia, Canada) and (5) angular (X-RSM40B-T4A) displacement stages, (6) a PD (PDA100A-EC, Thorlabs) attached to each RF output and an acquisition card (DAQ6510, Keithley, Solon, OH, USA) for digitizing the detector signals.

We tested four bifurcated fiber bundles with different fiber core sizes and numbers of fibers, plus one trifurcated bundle. Three of these bundles are constructed from just two fibers of diameters $\lbrace 50,200,600\rbrace$ µm. Their specs are given in Table 2. Additionaly, we included a randomly distributed bundle BF19Y2LS02, which has 19 high-grade fibers arranged in a round geometry. It splits into ends containing 9 TFs and 10 RFs, each having a 200 µm core and $\textrm{NA} = 0.22$. Compared to the simple two-fiber bundles, the 19-fiber design provides more uniform illumination- since the laser spot covers 9 TFs- and collects more total power with its 10 RFs. These bundles are SMA905-terminated.

**Table 2:** Specifications of the four bifurcated sensors employed for validation.

Bundle	BFY50LS02	BFY200LS02	BFY600LS02	BF19Y2LS02	Trifurcated
Bundle tip					
*R* ($\upmu$m)	135	245	650	*1	*2
NA (a.u.)	$0.22 \pm 0.02$	0.39	0.39	$0.22 \pm 0.02$	$0.22 \pm 0.02$
Diameter ($\upmu$m)					
Core	$50 \pm 1$	$200 \pm 5$	$600 \pm 10$	$200 \pm 4$	$200 \pm 4$
Cladding	$125 \pm 1$	$225 \pm 5$	$630 \pm 10$	$220 \pm 2$	$220 \pm 2$
Coating	$250 \pm 10$	$500 \pm 30$	$1040 \pm 30$	$320 \pm 16$	$320 \pm 16$

## Experimental outcomes and discussion

In this section we discuss the results corresponding to the bundles presented in Table 2.

### Bifurcated bundles

**Fig. 13:**
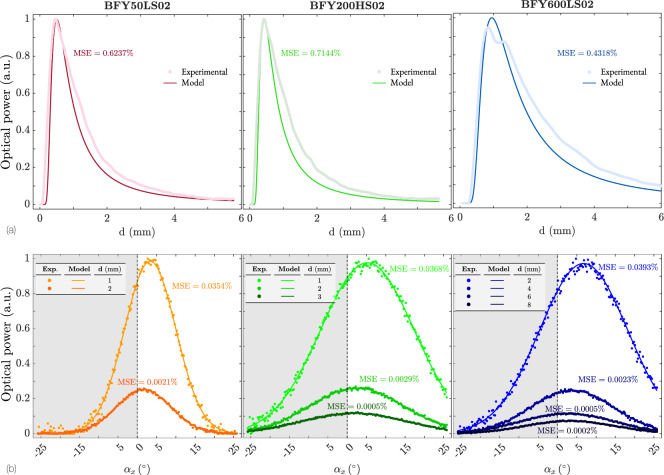
Experimental and theoretical responses of the three bifurcated two-fiber bundles with diameters $\lbrace 50,200,600\rbrace$ µm. (**a**) Responses as functions of distance for tilt $\alpha _x = 0^{\circ }$. (**b**) Response for fixed distances and $\alpha _x \in [-25, 25]^\circ$.

Figure 13a corresponds to the linear displacement measurements, while Fig. 13b relates to the angular displacement.

As a general observation, we can see that the developed theoretical model aligns very well with the experimental results. Regarding linear displacement, it is observed that the maximum power detected by each bundle shifts to higher distance values as the distance between the TFs and RFs increases.

The model predicts a steeper decrease after the maximum value, although in the end, for large distances, the experimental and theoretical power tends to the same asymptotic value.The differences at large distances primarily are due to two facts: First, at these distances, the reflected Gaussian beam expands beyond the fiber core and is partially clipped by the fiber cladding, resulting in a lower collected power. Second, excitation of higher-order modes in the TF distorts the beam profile from the ideal Gaussian assumed by the model, increasing divergence in the far field and thus reducing the coupling efficiency into the RF.

Figure 13b presents the measurements as a function of the angle for different distances. The first observation is an asymmetric response, as mentioned in Sec .3.1, meaning that the power reaches a maximum for positive angles. As the distance increases, the power decreases and becomes more symmetric, with the maximum closer to $\alpha _x = 0^\circ$. This occurs because the relative effect of the distance *R* between the RFs decreases with respect to the distance to the mirror, and is more steep when the size of the bifurcated bundle is smaller. For the 50 µm bundle, the response becomes symmetric at a distance of only 2 mm, whereas for the 600µm , symmetry is not achieved until a distance of 8 mm.

### Bifurcated 19-fiber bundle

**Fig. 14:**
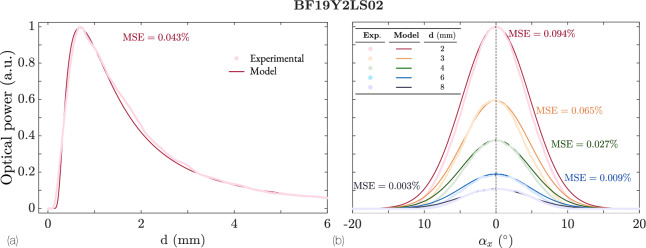
Experimental and theoretical responses of the 19 fibers bifurcated bundle BF19Y2LS02. (**a**) Response as a function of distance for $\alpha _x = 0^{\circ }$. (**b**) Response for five fixed distances and tilt angles $\alpha _x \in [-25, 25]^\circ$.

Figure 14 showcases the results corresponding to the bundle of 19 fibers. In this case, the fit between the theoretical model and the experimental data is even better. This could be attributed to the averaging effect of the 19 fibers, which mitigates individual misalignment. Due to the nearly random arrangement of the fibers and the more homogeneous structure of the beam emitted by the 19 fibers, the angular response is symmetric, meaning the maximum is centered at $\alpha _x = 0^\circ$. Unlike the previous case, no differences are observed between positive and negative angles, making this bundle a good candidate for angle measurements.

As expected, the sensitivity of the bundle increases inversely with distance. However, this bundle exhibits a faster loss of sensitivity with respect to angle; the response is minimal for $\alpha _x \notin [-10, 10]$. In any case, the response is bivalued, meaning this fiber arrangement cannot detect the direction of the angle of rotation.

### Trifurcated bundle

**Fig. 15:**
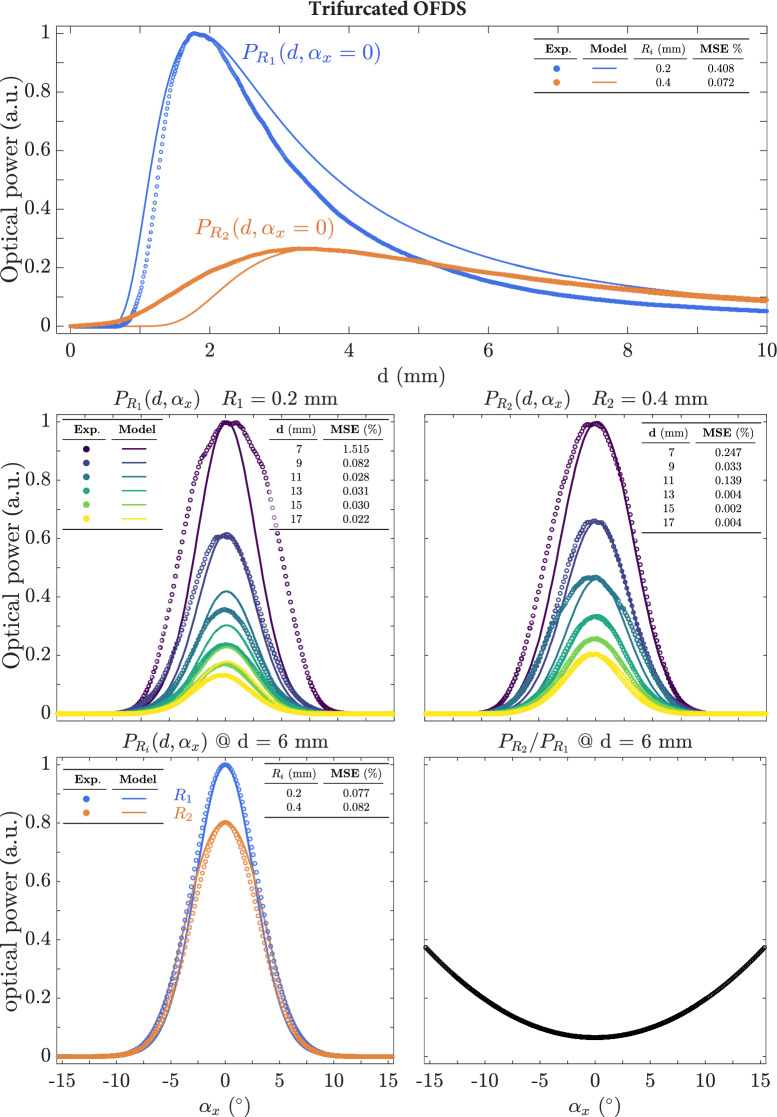
Experimental and theoretical responses of the two RFs collections $R_1$ and $R_2$ of the trifurcated bundle. (**a**) As a function of distance for $\alpha _x = 0^{\circ }$. (**b**) For fixed distances and tilt angles $\alpha _x\in \lbrace -15,15\rbrace ^\circ$. (**c**) Left, variying the tilt angle for a 6 mm distance for the two collections. Right, ratio of the received powers of the two collections with respect to the angle

The last case studied corresponds to a trifurcated bundle, from which we used two RFs collections to compare their experimental results with the model presented in Sec. 3.4. Figure 15 present the results for the distances of two RFs collections, located at $\lbrace R_1\approx 200,R_2\approx 400\rbrace$µm, respectively.

In this case, the fit is good, although not as precise compared to the previous bundles. This is since the model developed in Sec Trifurcated bundle is based on a linear fiber configuration, whereas the experimental results correspond to an azimuthal distribution. Despite this, the model fits quite well. The greatest discrepancy is observed in the value of the dead zone of the second RF collection, which is significantly larger in the theoretical model. This behavior can be explained by the fact that the RF collection project over shorter distances when considering a concentric structure, thereby reducing the dead zone.

Figure 15b shows the results as a function of the angle for the two RF collections. The normalized response is very similar for both RF collections, although, in absolute terms, the response of the first RF collection is greater. Nevertheless, the response of the first RF collection, located at $R_1$, does not fit the theoretical model as for the previous bundles. Specifically, the experimental response is broader and deteriorates for larger distances, for the same reasons outlined in the previous sections. In contrast, the model predicts the results for the second RF collection, located at greater distances, quite accurately. In both cases, the response is symmetric and centered at $\alpha _x=0^\circ$.

As described in Sec. Trifurcated bundle, this type of bundle can be used to achieve a noise-free response. For example, Fig. 15c presents the results for the two RFs collections at a distance $d=6$ mm. The response for the second RF collection is about 20% lower. By taking the ratio of the two powers, we obtain the results shown on the right side of the figure, where the response is plotted as a function of the angle. The response is low-noise and symmetric. However, the sensitivity decreases for smaller angles, which can be explained by the presence of the maxima in the individual responses of the RF collections.

All the previous results can be summarized in the following key points. The bundles generally provide reliable measurements of both distance and angle. Regarding angle measurement, the response is more symmetric for the 19-fiber bundle with a quasi-random structure and for the trifurcated bundle with azimuthal symmetry, although the latter exhibits less noise. However, while both bundles allow for accurate angle measurement, they are unable to determine the direction of the rotation axis of the mirror or target, nor can they distinguish the direction of rotation.

### Comparison with other work

We have also compared the proposed sensors with similar optical-fiber-based angular sensors reported in the literature. Table 3 summarizes key characteristics such as geometry, angular range, and nominal sensing distance.

One hallmark of our trifurcated optical fiber displacement sensor (OFDS) is that it combines several practical benefits into a single, compact device:

- A wide and linearized sensing range up to $15\,\textrm{mm}$.
- An angular range up to $\pm 15^\circ$.
- Comparable resolution of about 25 µm.
- A simple, reliable geometry that is easy to fabricate and miniaturize.

These features make it an excellent candidate for applications demanding compactness and reliability, such as monitoring aircraft engines. In brief, our design manages to sustain a good balance among angular range, distance range, and resolution while preserving simplicity in fabrication. That interplay between sensitivity, range, and geometry can be tuned further by choosing different fiber diameters or altering the NA.

**Table 3:** Comparison of sensor designs based on geometry, angular range, and distance.

Geometry	Angular range *x* (rad)	Angular range *y* (rad)	Distance (mm)	Other	References
Trifurcated	$\pm 15.0$	$\pm 15.0$	0.0, 15.0	–	Ours
Randomly distributed 19-fiber bundle	$\pm 15.0$	$\pm 15.0$	0.0, 1.0	–	^43^
T-shape	-	$\pm 0.1$	0.0, 2.0	–	^44^
Cross-shape	$\pm 0.1$	$\pm 0.1$	0.0, 2.0	–	^45^
Inclined fibers	-	-	0.0, 11.0	–	^28^
Bifurcated (2 fibers)	-	0.35–0.87	–	–	^46^
Line	-	$\pm 0.044$	0.0, 1.8	–	^33^
Symmetric bifurcated	-	$\pm 0.175$	–	–	^47^
Trapezoidal (4 fibers)	-	$\pm 0.4$	0.0, 2.0	Expensive. Complicated.	^34^
Bifurcated (2 fibers)	-	$\pm 0.175$	0.0, 2.0	Not differential	^48^
Bifurcated (2 fibers)	-	$\pm 0.0012$	32.0, 132.0	Not differential	^26^
Square (4 fibers)	$\pm 0.04$	$\pm 0.04$	0.0, 2.0	–	^49^
Symmetric trifurcated	-	$\pm 0.105$	0.0, 3.0	–	^50^
1 bundle + 2 inclined bundles (18 fibers)	-	$\pm 0.7$	0.5, 3.5	3 bundles	^51^
Bifurcated (2 fibers)	-	$\pm 0.126$	0.0, 1.0	Not differential	^52^
Bifurcated (2 fibers)	-	$\pm 0.023$	0.0, 2.0	–	^23^
Twisted macro–bend (2 fibers)	-	$0,2\pi$	0.0, 160.0	Bulky	^30,31^

**Table 4:** Advantages and limitations of the fiber bundles. The sense of rotation $\theta$ can be positive or negative. The omni-directional refers to the (*X*, *Y*) plane.

Layout	$\theta$	Omni	Slope (°^–1^)	Lin. range (mm)		Ang. range (°)		Drift (±5%)
				Min	Max	Min	Max	
Bifurcated	-	✗	5.6	0	6	0	20	NA 5.8%
Symmetric	+	✗	5.4	0	6	-15	15	NA 2.9%
Differential	+	✗	5.0	1	10	-15	15	NA 2.8%, R 3.8% (10°)
Trifurcated	+	✓	5.2	1	15	-10	10	NA 0.4% (0°) $\rightarrow$ 9% (10°)
Quasi-random	-	✓	5.4	0	6	-10	10	NA 4.9%

### Advantages and limitations of the different geometries

Sensor performance is governed not only by the analytic error bound in Sec. Sensitivity analysis but also by practical factors such as bundle size, ability to distinguish the sign of $\theta$ (sense of rotation), or to measure tilts on any axis, immunity to common-mode drift, and ease of calibration and fabrication.

Bifurcated bundles are mechanically the smallest and deliver the steepest power-–angle slope ($\approx 3.2\ \textrm{rad}^{-1}$ at $\alpha \approx 0^\circ$) but cannot identify the sign of the tilt and their output drifts most with $\textrm{NA}$ ($\pm 5.8\%$ for a $\pm 5\%$ NA change). It is noisy because it has only one RF.

A symmetric bifurcated pair distinguises the sense of rotation and halves the NA sensitivity, at the cost of doubling the fiber count. The differential variant, Eq. (17), cancels the common Gaussian prefactor. For this reason, the core–radius drift simplifies and $\textrm{NA}$ and $R$ contributions fall below $4\%$ even at $\alpha =10^\circ$, making it the most stable of the single–axis bundles. All these bundles are restricted to single–axis angular measurements.

The trifurcated design, Eq. (19), suppresses power noise and decouples distance from angle, enabling simultaneous $\alpha$ and $d$ extraction, but NA drift re–enters at large tilt ($\pm 9\%$ at $\alpha =10^\circ$) and the $1.9\ \textrm{mm}$ tip diameter is the largest of the fiber bundles.

Finally, the quasi-random 19-fiber bundle, Eq. (22), averages over ten TF–-RF separations, producing an almost perfectly symmetric response for any azimuth and keeping every $\pm 5\%$ tolerance below $5\%$. Its main limitation is the mechanical assembly complexity and noise, as well as its low sensitivity at small tilts. The last two designs can measure rotations around X and Y axis.

## Conclusions

We developed and experimentally validated a unified analytical model for intensity-based optical fiber angle sensors (OFASs) capable of measuring target tilt about one or more orthogonal axes. By incorporating Gaussian beam propagation, geometry, and reflection constraints, our approach analytically links measured power to tilt angles, obviating the need for purely empirical calibrations.

We examined configurations from simple bifurcated two-fiber designs to symmetric, differential trifurcated, and randomly distributed 19-fiber bundles. Notably, geometric symmetry and differential detection greatly reduce noise and measurement ambiguity. Experimental validation over distances up to 15 mm and tilt angles up to $\pm 20^\circ$ confirms that the model accurately captures both the angular and distance-dependent sensor behavior. Bundles with higher fiber counts exhibit stable, less ambiguous responses, demonstrating clear advantages for reliable multi-axis sensing.

Compared to existing optical-fiber-based angular sensors, our model offers three key strengths: (1) a closed-form power expression enabling straightforward tilt inference, (2) flexible adaptation to different fiber arrangements, and (3) enhanced noise rejection through differential detection. These features support compact, reliable, multi-axis angle measurements suitable for harsh or space-constrained environments, spanning industrial inclinometers to aerospace components. Our trifurcated design captures all this features, as it was demonstrated both theoretical and experimentally.

The $\pm 5\%$ sensitivity analysis demonstrates that even the worst tolerance ($-5\%$ NA), changes the received power by just $5.8\%$, corresponding to a $<0.30^\circ$ additional tilt error. This confirms that standard-spec fibers are sufficient for correct sensor performance in practical scenarios.

Future work will extend the approach to larger angular ranges, non-specular targets, and dynamic conditions. Ongoing efforts to integrate real-time data processing may improve resolution and reduce hardware complexity. Our results highlight multi-fiber intensity-based sensing as a promising technology for Industry 5.0, offering durable, cost-effective, and axis-resolved angle measurements in diverse, demanding applications.

## Data Availability

The datasets used and/or analyzed during the current study are available from the corresponding author on reasonable request.

## Appendix

We start from the exact 1-D form of the collected power for one RF (already after integrating over $x$):

23 $$\begin{aligned} P_{\textrm{exact}} =\int _{R - r}^{R + r}F(y)\,\textrm{d}y \end{aligned}$$

where,

24 $$\begin{aligned} F(y) = A\,{{\,\textrm{erf}\,}}\!\Bigl (\frac{\sqrt{2\bigl (r^2 - (y - R)^2\bigr )}}{\omega }\Bigr ) \exp \!\bigl [-\,a\,(y\cos 2\alpha - d\sin 2\alpha )^2\bigr ]. \end{aligned}$$

The closed-form approximation in Eq. (9) uses

25 $$\begin{aligned} P_{\textrm{approx}} = 2r\,F(R). \end{aligned}$$

Because $F(y)$ is smooth on $[R - r,\,R + r]$ we may write the exact integral as,

26 $$\begin{aligned} P_{\textrm{exact}} = 2r\,{\overline{F}}, \quad {\overline{F}} = \frac{1}{2r}\,\int _{R - r}^{R + r} F(y)\,\textrm{d}y. \end{aligned}$$

Hence, the relative error is calculated as

27 $$\begin{aligned} \varepsilon = \frac{\bigl |P_{\textrm{approx}} - P_{\textrm{exact}}\bigr |}{P_{\textrm{exact}}} = \frac{|F(R) - {\overline{F}}|}{{\overline{F}}}, \end{aligned}$$

so, we need to compare the *center* value $F(R)$ with the *interval average* ${\overline{F}}$. Let $y = R + u$ with $u\in [-r,+r]$. A second-order Taylor series gives

28 $$\begin{aligned} F(R + u) = F(R) + F'(R)\,u + \tfrac{1}{2}\,F''(R)\,u^2 + O(u^3). \end{aligned}$$

Since the interval $[-r,r]$ is *symmetric*, the odd term $F'(R)$ integrates to zero, so the average becomes

29 $$\begin{aligned} {\overline{F}} =F(R)+\tfrac{1}{2}\,F''(R)\,\frac{1}{2r}\int _{-r}^{r}u^2\,du + O(u^4). \end{aligned}$$

Therefore,

30 $$\begin{aligned} F(R)-{\overline{F}} =-\frac{r^2}{6}\,F''(R) + O(u^4). \end{aligned}$$

Near $y=R$, the error-function term ${{\,\textrm{erf}\,}}(\cdots )\approx 1$, so to leading order

31 $$\begin{aligned} F(y)\approx A\exp [-\,a\,(y\cos 2\alpha - d\sin 2\alpha )^2] \end{aligned}$$

and deriving twice,

32 $$\begin{aligned} F''(R) =\Bigl (\frac{4R^2}{\omega ^4}-\frac{2}{\omega ^2}\Bigr )\,F(R) \end{aligned}$$

inserting that into Eq. (30):

33 $$\begin{aligned} \bigl |F(R)-{\overline{F}}\bigr |=\frac{r^2}{6}\Bigl |\frac{4R^2}{\omega ^4}-\frac{2}{\omega ^2}\Bigr |\,F(R) +O(u^4). \end{aligned}$$

Because $R\gg r$ (600 µm vs 50 µm) the first term inside the brackets is negligible,

34 $$\begin{aligned} \bigl |F(R)-{\overline{F}}\bigr |\approx \frac{r^2}{3\,\omega ^2}\,F(R), \end{aligned}$$

and plugging into Eq. (27) and taking into account that ${\overline{F}}\le F(R)$:

35 $$\begin{aligned} \varepsilon (d,\alpha ) =\frac{|P_{\textrm{approx}}-P_{\textrm{exact}}|}{P_{\textrm{exact}}} =\frac{|F(R)-{\overline{F}}|}{{\overline{F}}} \le \frac{r^2}{\omega ^2}+O\bigl (\tfrac{r^4}{\omega ^4}\bigr ). \end{aligned}$$

In summary, small RF ($r$) or broad spot ($\omega$) means that the quadratic term $r^2/\omega ^2$ is tiny, so Eq. (9) is very accurate. Large spacing $R$ only appears in the neglected $4R^2/\omega ^4$ term, and is suppressed by $\omega ^2$; at $d\le 8$ mm the $R$-dependent part contributes $<0.2\%$ to $\varepsilon$. The expansion shows explicitly why $\varepsilon$ shrinks when NA (and therefore $\omega$) increases, exactly as the numeric sweep confirms.
